# NT1-Tau Is Increased in CSF and Plasma of CJD Patients, and Correlates with Disease Progression

**DOI:** 10.3390/cells10123514

**Published:** 2021-12-13

**Authors:** David Mengel, Tze How Mok, Akin Nihat, Wen Liu, Robert A. Rissman, Douglas Galasko, Henrik Zetterberg, Simon Mead, John Collinge, Dominic M. Walsh

**Affiliations:** 1 Laboratory for Neurodegenerative Research, Ann Romney Center for Neurologic Diseases, Brigham and Women’s Hospital and Harvard Medical School, Boston, MA 02115, USA; david.mengel@uni-tuebingen.de (D.M.); wliu3@bwh.harvard.edu (W.L.); 2 Research Division “Translational Genomics of Neurodegenerative Diseases”, Hertie-Institute for Clinical Brain Research and Center of Neurology, University of Tübingen, 72076 Tübingen, Germany; 3 German Centre for Neurodegenerative Diseases (DZNE), 72076 Tübingen, Germany; 4 MRC Prion Unit at UCL, UCL Institute of Prion Diseases and NHS National Prion Clinic, National Hospital for Neurology and Neurosurgery, UCL Hospitals NHS Foundation Trust, London W1W 7FF, UK; tze.mok@ucl.ac.uk (T.H.M.); akin.nihat@nhs.net (A.N.); s.mead@prion.ucl.ac.uk (S.M.); jc@prion.ucl.ac.uk (J.C.); 5 Department of Neurosciences, University of California San Diego, La Jolla, CA 92093, USA; rrissman@ucsd.edu; 6 VA San Diego Healthcare System, La Jolla, CA 92161, USA; dgalasko@ucsd.edu; 7 Department of Neurodegenerative Disease, UCL Institute of Neurology, Queen Square, London WC1N 3BG, UK; h.zetterberg@ucl.ac.uk; 8 UK Dementia Research Institute at UCL, London WC1E 6BT, UK; 9 Clinical Neurochemistry Laboratory, Sahlgrenska University Hospital, S-431 80 Mölndal, Sweden; 10 Department of Psychiatry and Neurochemistry, Institute of Neuroscience and Physiology, the Sahlgrenska Academy at the University of Gothenburg, S-431 80 Mölndal, Sweden; 11 Hong Kong Center for Neurodegenerative Diseases, Hong Kong, China

**Keywords:** Alzheimer’s disease, biomarker, blood, cerebrospinal fluid, neurodegeneration, neurofilament light chain, prion disease, Simoa-immunoassays

## Abstract

This study investigates the diagnostic and prognostic potential of different forms of tau in biofluids from patients with Creutzfeldt-Jakob disease (CJD). Extracellular tau, which is molecularly heterogeneous, was measured using ultra-sensitive custom-made Simoa assays for N-terminal (NT1), mid-region, and full-length tau. We assessed cross-sectional CSF and plasma from healthy controls, patients with Alzheimer’s disease (AD) and CJD patients. Then, we evaluated the correlation of the best-performing tau assay (NT1-tau) with clinical severity and functional decline (using the MRC Prion Disease Rating Scale) in a longitudinal CJD cohort (*n* = 145). In a cross-sectional study, tau measured in CSF with the NT1 and mid-region Simoa assays, separated CJD (*n* = 15) from AD (*n* = 18) and controls (*n* = 21) with a diagnostic accuracy (AUCs: 0.98–1.00) comparable to or better than neurofilament light chain (NfL; AUCs: 0.96–0.99). In plasma, NT1-measured tau was elevated in CJD (*n* = 5) versus AD (*n* = 15) and controls (*n* = 15). Moreover, in CJD plasma (*n* = 145) NT1-tau levels correlated with stage and rate of disease progression, and the effect on clinical progression was modified by the *PRNP* codon 129. Our findings suggest that plasma NT1-tau shows promise as a minimally invasive diagnostic and prognostic biomarker of CJD, and should be further investigated for its potential to monitor disease progression and response to therapies.

## 1. Introduction

Creutzfeldt-Jakob disease (CJD) is the most common prion disorder afflicting humans and is characterized by rapid and widespread neurodegeneration [1,2,3,4]. Aggregates of tau are a prominent feature of several neurodegenerative diseases [5,6] and an accumulation of phosphorylated tau akin to that seen in Alzheimer’s disease (AD) has been reported in certain cases of CJD [7]. Elevated levels of tau in cerebrospinal fluid (CSF) are also seen in CJD and AD [8,9,10,11,12,13,14,15,16], but, unlike neurofilament (NfL), an elevation of CSF tau is not seen in most other neurodegenerative conditions [12,17,18].

An assessment of CSF tau using mid-region (MR) assays differentiates CJD from other neurodegenerative conditions (including rapidly progressing AD) and controls [8,9,12,13,15,19]. Indeed, measurements of MR-tau in CSF achieved sensitivities (75–98%) and specificities (67–99%) for CJD that exceeded the diagnostic performance of the accepted clinical marker 14-3-3 [15]. However, the use of a single immunoassay provides little information about which forms of tau are elevated in CJD. This is important since increasing evidence suggests that extracellular tau is molecularly complex and includes an array of differentially truncated forms of tau, some of which may have greater diagnostic potential than others [20,21,22,23,24,25,26,27]. Thus, measuring different forms of tau in human fluids not only affords an opportunity to identify and evaluate new biomarkers, but should provide insights into the forms of tau involved in disease and their molecular mechanisms.

Here, we measured tau in CSF using three well-validated assays: a novel assay, which employs antibodies with epitopes identical to those used in the widely applied Innotest assay, and requires a mid-region sequence of 194–224; the NT1 assay, which detects forms of tau that contain the minimal sequence of 6–198 [28]; and an assay that employs antibodies to the extreme N- and C-termini of tau and is specific for full-length protein [20]. When used together, these assays provide information about the relative abundance of FL-tau, N-terminal and mid-region fragments.

Biofluids from patients with CJD potentially contain infectious prions. To enable the analysis of CJD samples in a standard research laboratory setting we optimized our assays for use with samples treated with prion-destroying concentrations of guanidine hydrochloride (GuHCl). In accordance with our prior findings [20], mid-region and NT1 assays measured the highest levels of tau in CSF, and we discovered that the NT1-tau assay perfectly discriminated CJD from both AD and controls. Applying some of the same ultrasensitive assays to plasma revealed that NT1-detected tau is increased in CJD versus controls and AD. Importantly, NT1-tau levels were associated with functional decline and rate of clinical progression, and a known modifier of CJD phenotype, polymorphism of the prion gene (*PRNP*) at codon 129, influenced the rate of clinical progression [29,30,31,32].

## 2. Materials and Methods

### 2.1. Participants

Demographics and clinical information about cases and controls are provided in Supplemental Table S1.

#### 2.1.1. CJD Study Cohorts

CJD cases were from the UK National Prion Monitoring Cohort study (NPMC) [33] and/or the PRION-1 trial [34]. CSF samples were from 15 CJD patients (13 sporadic and 2 iatrogenic) and these, together with 18 AD and 21 healthy control specimens (collected in Gothenburg), are referred to as *Study 1* samples. Plasma samples from six of the same CJD patients in *Study 1*, together with 15 control and 15 AD specimens collected at UCSD, constitute *Study 2* samples. Plasma samples collected from 145 sporadic CJD patients included 117 patients from whom plasma was taken at a single timepoint (*Study 3*) and 28 patients from whom plasma specimens were obtained at two or more different timepoints (*Study 4*).

All CJD patients included in this study were classified as either definite or probable CJD according to published criteria [35]. Patients were systematically assessed at enrollment and assessed at intervals of 6–8 weeks. Informed consent was obtained from all subjects involved in the study. Specifically, research consent was obtained according to a protocol reviewed by the Scotland A Research Ethics Committee (NPMC) or the Eastern Research Ethics Committee (PRION-1). Functional status, and hence disease severity, was measured using the Medical Research Council Prion Disease Rating Scale (MRC Scale), which ranges from 20 (no significant impairment) to 0 (severely impaired, bedbound, unable to communicate or swallow) [33]. A slope coefficient representing the percentage loss of function per day for individual patients was calculated [29].

Blood was collected in EDTA-coated tubes and centrifuged at 2000× *g* for 15 min. Plasma was aliquoted into 1.5 mL polypropylene tubes and stored at −80 °C. Lumbar CSF was collected into polypropylene tubes and processed within 1 h. Samples were centrifuged at 2200× *g* for 10 min, aliquoted into 1.5 mL polypropylene tubes and stored at −80 °C. Prior to shipment to the Walsh lab, samples were thawed and treated with 5 M GuHCl to denature infectious prion species and then transported on dry ice.

#### 2.1.2. AD and HC Study Cohort

CSF specimens came from AD and control cases (see Supplemental Table S1, CSF study cohort 1) recruited at the University of Gothenburg [36] under the approval of the local ethics committee. CSF was collected, processed, and stored as in Section 2.1.1. Patients were designated as normal or AD according to CSF biomarker levels using cutoffs that are 90% specific for AD [37]: tau > 350 pg/mL and Aβ42 < 530 pg/mL [38,39].

Plasma samples from AD and control subjects (Supplemental Table S1, plasma study cohort 1) were collected at the Shiley-Marcos Alzheimer’s Disease Research Center (ADRC) under a protocol approved by the UCSD Ethics Committee. Blood was collected, processed, and stored as in Section 2.1.1. Control subjects had an MMSE score ≥ 28, tau/Aβ1-42 ratio < 0.5, and Aβ1-42 concentration > 630 pg/mL. AD subjects had a tau/Aβ1-42 ratio > 0.88 and Aβ1-42 ≤ 630 pg/mL and an MMSE score of 15–24 points.

To enable a comparison of measurements made in CJD versus AD and control specimens, samples from Gothenburg and UCSD were treated with 5 M GuHCl prior to analysis.

### 2.2. CSF and Plasma Analysis

#### 2.2.1. Homebrew Simoa-Based Assays for N-Terminal and Full-Length Tau

Validation of the N-terminal (NT1) and the full-length (FL) tau assays were reported previously [20,28]. The lower limit of quantitation (LLoQ) was defined as the lowest standard: (i) with a signal higher than the average signal for the blank plus 9 SDs, and (ii) allows a percent recovery ≥100 ± 20%. The LloQs (in the presence of 0.25 M GuHCl) for the NT1 and FL-tau assays were 0.74 pg/mL. The repeatability of the NT1-tau and FL tau assays for two internal control samples was determined as 8.2% and 6.9%, and 10.0% and 2.5%, respectively. Measurements of NT1-tau in 4 plasma samples (1.8% of all specimens) produced inter-assay CVs > 20% (between initial and repeated analysis), and these samples were excluded from further analysis.

#### 2.2.2. Homebrew Simoa-Based Assay for Mid-Region Tau

Conjugation of beads with BT2 (194–198; Thermo, Waltham, MA, USA) and biotinylation of the detector antibody ADx202 (218–224; ADx Neurosciences, Gent, Belgium) were carried out as described previously [28] and the assays were run using a 2-step procedure. The MR-tau assay was highly sensitive to GuHCl and samples had to be diluted 1:40 to a final concentration of 0.063 M GuHCl. The LLoQ in the presence of 0.063 M GuHCl was 6.7–20 pg/mL. The repeatability of the MR-tau assay for two internal control samples was determined as 6.7% and 10.7%.

#### 2.2.3. NfL

Simoa™ NF-light^®^ Advantage (Quanterix, Billerica, MA, USA) kits were used according to the manufacturer’s instructions. Samples pre-treated with GuHCl were processed as for the NT1- and FL-tau assay except NfL diluent was used. The LLoQ in the presence of 0.25 M GuHCl was 1.39–1.56 pg/mL. The repeatability of the NfL assay for two internal control samples was determined as 4.0% and 3.3%.

### 2.3. Statistical Analysis

Statistical analyses were carried out using GraphPad Prism, version 8 (LaJolla, CA, USA) and Stata, version 15.1 (Stata Corp., College Station, TX, USA). Differences in biomarker levels between groups were assessed using Kruskal–Wallis H test followed by Dunn’s post hoc test. Normal distribution was assessed by Shapiro–Wilk test and visual inspection of histograms and Q-Q-blots. Diagnostic accuracy was investigated using receiver operating characteristic (ROC) curve analysis with a non-parametric approach. Linear regression fits were used to study the association of plasma log_2_NT1 tau levels with severity of functional impairment (MRC Scale) and the rate of clinical progression (MRC slope), including an interaction term for the independent variable PRNP codon 129 genotype. Models were adjusted for gender and age at blood collection. Visual inspection of scatterplots indicated a linear relationship between variables. There was homoscedasticity (assessed with visual inspection of residual plots, Cameron and Trivedi’s decomposition of IM-test, and Breusch–Pagan test) and normality (assessed with visual inspection of histograms, Kernel density estimates, P-P-plots, and Q-Q-plots) of the residuals. For analysis of repeated measures, linear mixed-effect (LME) models were fitted to test whether stage of progression (MRC Scale) was associated with plasma NT1 tau levels in *PRNP* codon 129 MV cases. From a total of 28 patients in the longitudinal sub study (see Supplemental Table S1), 1 produced plasma NT1tau measurements with a CV > 20% and 3 had no MRC Scale available at time of blood draw, yielding a total of 24 patients available for longitudinal analysis. The LME models were adjusted for age and gender, and included random effects and intercepts nested within subject. For measurements that were below the LLoQ of the respective assay (*Study 1*: CSF FL tau, HC/AD groups 19/39; *Study 2*: Plasma NfL, HC/AD groups 27/30; Plasma NT1 tau, HC/AD groups 24/30; Plasma FL tau, HC/AD groups 23/30; *Study 3/4*: Plasma NT1 tau, CJD 8/188), statistical analysis was repeated after samples had been assigned values equal to the LLoQ of the assay but produced similar results. The significance threshold was set to a two-sided *p* ≤ 0.05.

## 3. Results

### 3.1. NfL, NT1- and FL-Tau Assays, but Not the Innotest MR-Tau ELISA, Are Compatible with Concentrations of GuHCl Which Allow Detection of Tau in Human CSF

**Figure 1:**
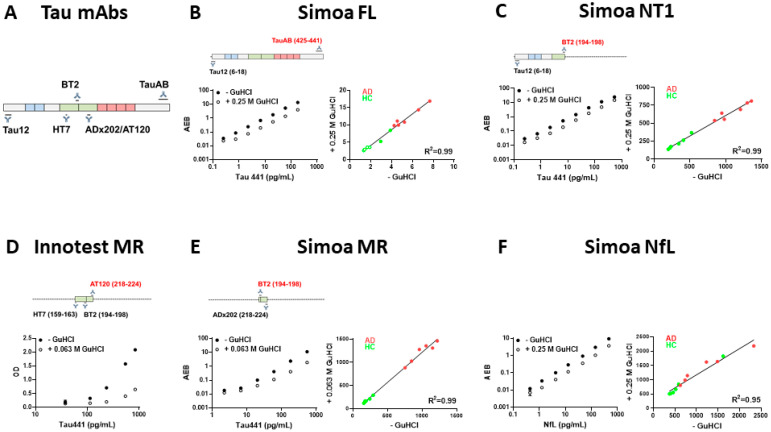
**The sensitivity of the NfL, FL-tau, and NT1-tau assays are similar in the presence/absence of 0.25 M GuHCl.** (**A**) Schematic representation of tau 441, and epitopes of antibodies used in each tau assay. Standard curves in the presence and absence of GuHCl for (**B**) Simoa FL-tau (LLoQ in the absence of GuHCl 0.25 pg/mL, LLoQ in the presence of 0.25 M GuHCl 0.74 pg/mL), (**C**) Simoa NT1-tau (LLoQ in the absence of GuHCl 0.25 pg/mL, LLoQ in the presence of 0.25 M GuHCl 0.74 pg/mL), (**D**) Innotest MR-tau ELISA (LLoQ in the absence of GuHCl 30 pg/mL, LLoQ in the presence of 0.063 M GuHCl 540 pg/mL), (**E**) Simoa MR (LLoQ in the absence of GuHCl 6.67 pg/mL, LLoQ in the presence of 0.063 M GuHCl 20 pg/mL), and (**F**) NfL (LLoQ in the absence of GuHCl 0.51 pg/mL, LLoQ in the presence of 0.25 M GuHCl 1.6 pg/mL). Each datapoint is the average ± SEM from a triplicate measurement. Where the error bars are not visible, the SEM is smaller than the size of the symbol. When appropriately diluted, GuHCl treatment did not alter detection of analytes in CSF specimens from 5 HC and 5 AD subjects (B, C, E and F right panels)-green circles are HC and red circles are AD patients (R^2^ = 0.95–0.99). Open green circles indicate measurements below the LLoQ. Note: the dramatic reduction in sensitivity of the MR Innotest caused by GuHCl precluded measurement of tau in CSF.

Biofluids from CJD patients have the potential to contain infectious prions and were treated with an agent known to abolish infectivity prior to transport and assay. Here, we tested compatibility with GuHCl of three established tau assays, a novel tau assay, and a commonly used assay for NfL. The NfL, NT1 and FL-tau assays retained their high sensitivity at GuHCl concentrations up to 0.25 M. Specifically, standard curves generated in the presence of 0.25 M GuHCl were comparable to those obtained in the absence of GuHCl (Figure 1B,C,F). In contrast, the presence of GuHCl greatly reduced the sensitivity of the widely used Innotest hTau Ag ELISA (Figure 1D). The reduced sensitivity and the requirement to dilute samples forty-fold (from 2.5 M to 0.063 M GuHCl) rendered it impossible to use the Innotest assay to measure tau in GuHCl-treated CSF. To overcome this problem, we developed and validated a Simoa-based MR assay that was ~27 times more sensitive than the Innotest MR assay. Although our MR Simoa assay also had low tolerance for GuHCl (Figure 1E), the increased sensitivity made it possible to dilute samples to abrogate the effects of GuHCl and still detect accurate values (Figure 1E, Supplemental Figure S1).

Importantly, when CSF from controls and AD subjects were analyzed in the presence or absence of 0.25 M GuHCl (0.063 M GuHCl for MR-tau), the values obtained were highly correlated (R^2^ ≥ 0.95). Thus, when GuHCl-treated samples are appropriately diluted, it is possible to accurately measure NfL and various forms of tau (Figure 1B,C,E,F).

### 3.2. CSF NfL and Distinct Species of Tau Differentiate between Controls, AD and CJD

**Figure 2:**
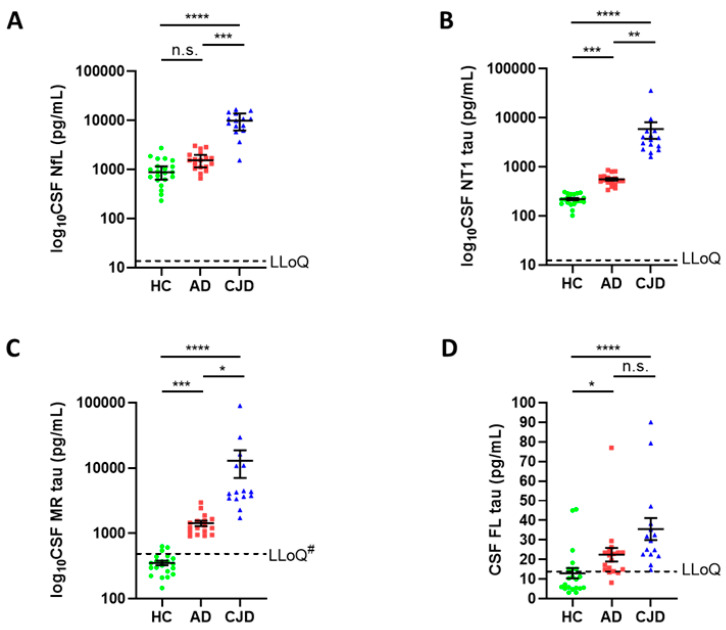
**CSF NfL and forms of tau measured with NT1, mid-region and full-length assays are increased in CJD compared to AD and controls.** *Study 1* CSF samples comprise 21 HC (green circles), 18 AD (red squares) specimens collected in Gothenburg, and 15 CJD (blue triangles) samples collected at UCL. All samples were treated with GuHCl, diluted as required and analyzed with Simoa-based assays for (**A**) NfL, (**B**) NT1-tau (BT2-Tau12), (**C**) MR-tau (BT2-ADx202), and (**D**) FL-tau (TauAB-Tau12). Each point represents a single individual and means ± SEM are indicated. Differences between groups were assessed with Kruskal–Wallis H test followed by Dunn’s post-hoc test. CSF NT1, MR, and FL-tau was elevated in AD versus controls, whereas NfL only showed a trend towards elevation in AD compared to controls. CSF levels of NfL, NT1, and MR-tau were strongly increased in CJD versus AD and controls, whereas FL-tau levels were only modestly higher in CJD. #MR-tau levels in CJD, AD and NC samples were measured in the presence of 0.063 M GuHCl, but samples were diluted 1:4 for NC and AD, and 1:80 for CJD samples. Therefore, the actual LLoQ for the AD and NC samples was ~27 pg/mL versus 536 pg/mL for the CJD samples. One CJD patient and one AD patient had MR-tau measurements above the upper limit of quantitation. Abbreviations: AD, Alzheimer’s disease; CJD, Creutzfeldt-Jakob disease; CSF, cerebrospinal fluid; FL-tau, full-length tau assay; MR-tau, mid-region tau assay; NT1-tau, N-terminal tau assay type 1; SEM, standard of the mean; n.s. non-significant; * *p* < 0.05; ** *p* < 0.01; *** *p* < 0.001; **** *p* < 0.0001.

Next, we investigated the forms of tau present in CSF from patients with CJD, whether they differed from forms of tau detected in AD and controls, and their relationship to a non-specific marker of neurodegeneration, NfL. To enable a comparison of absolute amounts of tau detected by different assays, the same recombinant tau standard was used for each. In CSF from controls, the mid-region assay detected the highest signal (352 ± 28 pg/mL) with the signals from the NT1 and FL assays, accounting for ~60% (220 ± 12 pg/mL) and ~4% (13 ± 3 pg/mL) of that detected by the MR assay. The levels of tau detected by each of the three assays were elevated in AD versus controls, in CJD versus controls, and in CJD versus AD (Figure 2). The average fold increase in CSF tau levels in AD and CJD versus HC was highest for MR-tau (AD versus HC 4.1, CJD versus HC 36.6), followed by NT1-tau (AD versus HC 2.6, CJD versus HC 26.8), and lowest for FL-tau (AD versus HC 1.7, CJD versus HC 2.7). These results are consistent with our earlier studies comparing different forms of tau in CSF from controls and AD patients [20], i.e., tau detected by mid-region assays account for the highest levels of tau in AD CSF, NT1-tau detects comparable but lower amounts of tau than MR assays, and FL-tau, although elevated in AD, accounts for only a fraction of the tau detected by the other two tau assays.

NfL is an accepted marker of neurodegeneration [40] and, as expected for an aggressive neurodegenerative disease such as CJD, NfL levels in CJD CSF were almost nine times higher than in controls (Figure 2A). Reflective of the more chronic course of neurodegeneration seen in AD [41], CSF NfL were only modestly elevated in AD versus HC (Figure 2A).

These results imply that the elevation of tau in CJD and AD CSF is driven not only by neurodegeneration, but by disease processes independent of neuronal death [23,42]. While the biological reasons for elevations of NT1- and MR-detected forms of tau are unclear, measurement of these analytes in CSF allow for a perfect or near-perfect separation of CJD patients from controls (AUC = 1 for both NT1-tau and MR-tau) and AD subjects (AUC = 1 for NT1-tau, and 0.98 for MR-tau, Supplemental Figure S2), and is slightly better than that obtained with NfL (Supplemental Figure S2). Even measurement of FL-tau in CSF permits a reasonable differentiation between diagnostic groups (AUCs ≥ 0.77). Forms of tau in CSF measured with the mid-region tau assay, are highly correlated with tau detected with the NT1-tau assay, but not the full-length tau assay (Supplementary Figure S3).

### 3.3. Plasma NfL and NT1-Tau Are Elevated in CJD Compared to AD and Controls

**Figure 3:**
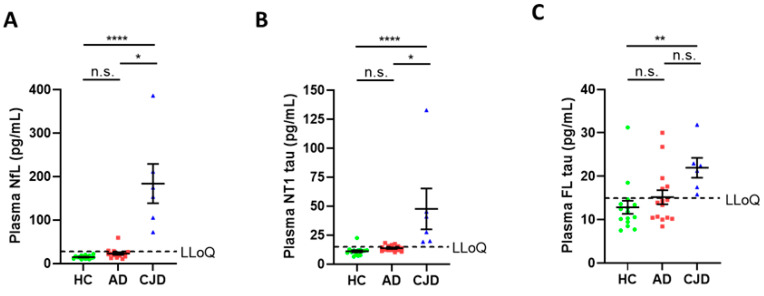
**Plasma NfL, NT1-tau and FL-tau are elevated in CJD compared to HC and AD subjects.** *Study 2* samples included plasma from 15 HC (green circles) and 15 AD (red squares) subjects collected at UCSD, and from 6 CJD (blue triangles) subjects collected at UCL. All samples were treated with GuHCl, diluted appropriately, and analyzed with Simoa-based assays for (**A**) NfL, (**B**) NT1-tau, and (**C**) FL-tau. Each point represents a single individual and means ± SEM are indicated. Differences between groups were assessed with Kruskal–Wallis H test followed by Dunn’s post-hoc test. Plasma levels of NfL, NT1, and FL-tau were elevated in CJD compared to controls and AD subjects. n.s. non-significant; * *p* < 0.05; ** *p* < 0.01; **** *p* < 0.0001. Note: because of the 10-fold dilution required to mitigate interference by GuHCl many AD and control values were below the reliable limit of quantitation; in contrast, when AD and HC samples were analyzed without addition of GuHCl NfL, NT1-tau and FL-tau were readily detected (see Supplemental Figure S4).

Given the encouraging results seen in CSF, we expanded our study to investigate plasma. Since the MR Simoa assay had a relatively high LLoQ and greater sensitivity to GuHCl analysis, this assay was not used to measure tau in plasma. Even for the NfL, NT1 and FL-tau assays, many AD and control plasmas, which, when unmanipulated, had readily measurable values (Supplemental Figure S4), did not yield detectable signals when treated with GuHCl (Figure 3). This was because the 20-fold dilution required to accommodate GuHCl treatment often brought tau levels below the LLoQs of the assays. In contrast, the high dilution required to accommodate GuHCl did not prevent the detection of NfL, NT1-tau and FL-tau in CJD samples (Figure 3A–C).

### 3.4. NT1-Tau Levels Are Associated with Clinical Progression of CJD

**Figure 4:**
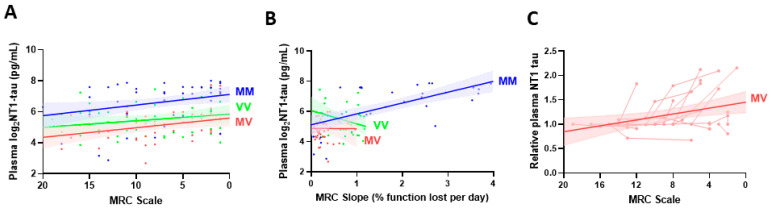
**Plasma NT1-tau levels positively correlate with disease progression in CJD subjects.** Samples from *Study 3* and baseline samples from *Study 4* comprised plasma specimens from 145 CJD patients. Association of plasma NT1-tau levels with (**A**) severity of functional impairment measured with the MRC Scale, and (**B**) the rate of clinical progression determined with the MRC Slope was modelled using linear regression stratified by (PRNP) polymorphism at codon 129. (**A**) Severity of functional impairment (lower MRC Scale) correlated with higher plasma log_2_NT1-tau levels in CJD patients (Supplemental Figure S5A). Plasma log_2_NT1-tau levels were higher in MM (blue) vs. MV (red) and VV cases (green) (*p* < 0.001), but the slopes were not significantly different between codon genotypes (MRC scale*Codon129 interaction: *p* = 0.835). (**B**) A faster rate of clinical progression (greater MRC Slope) was associated with increased levels of log_2_NT1-tau in MM (blue), but not in MV (red) and VV cases (green) (MRC slope*Codon129 interaction: *p* = 0.012). (**C**) Study 4 samples were from 17 MV CJD cases and included a total of 46 longitudinal plasmas. Fold changes in repeated NT1-tau measures were calculated relative to the measurement at initial visit, and are expressed as relative plasma NT1-tau levels. Association of plasma NT1-tau levels with MRC Scale was modelled using a linear mixed effects regression. Shaded areas represent 95% confidence intervals. Spaghetti plots show repeated measures for individual subjects. Relative plasma NT1-tau levels increase moderately in MV cases (*p* = 0.008). Repeated measures were only available from a limited number of MM (*n* = 2) and VV (*n* = 5) cases, and are shown in Supplemental Figure S5C.

Having shown that NT1-tau can readily be detected in plasma from patients with CJD, and at levels elevated versus AD and HC (Figure 3), we next investigated whether NT1-tau levels change with the progression of CJD. First, we conducted a cross-sectional analysis of 145 CJD individuals across a spectrum of disease stages (including Study 3 samples and baseline samples from Study 4; see Supplemental Table S1). We examined the relationship between plasma NT1-tau levels and two parameters, MRC Scale (Figure 4A) and MRC slope (Figure 4B). The 20-point MRC Scale has been shown to capture progression across the full range of physical and cognitive domains that are affected by CJD [33]. MRC scale is a snapshot of the clinical status of a patient at the time of sampling, and the MRC slope provides a measure of the rate of disease progression throughout the observed disease period. Stage of disease based on the MRC scale was a predictor of higher log_2_NT1-tau levels in CJD patients (β = −0.07, *p* < 0.001) (Supplemental Figure S5A). Log_2_NT1-tau levels were higher in MM vs. MV and VV carriers (*p* < 0.001), but changed at a similar rate with increasing MRC scale between codon 129 carriers (MRC-Scale*Codon129 interaction: p = 0.835) (Figure 4A). Rate of disease progression based on the MRC slope correlated with log_2_NT1-tau levels, but the effect was modified by the PRNP codon 129 genotype (Supplemental Figures S5B and Figure 4B). Higher log_2_NT1-tau levels predicted steeper MRC slopes in MM patients (MRC-Slope, β = 0.72), but not in MV (β = −0.03) and VV cases (β = −0.87) (MRC slope*Codon129 interaction: *p* = 0.012) (Figure 4B).

Next, we investigated longitudinal samples from 24 CJD patients for which MRC Scale scores were available at the time of blood collection. When sampled at a timepoint with a lower MRC Scale score versus baseline, plasma NT1-tau levels were increased in 16 patients, 3 were unchanged, and 5 had decreased NT1-tau concentrations (Supplemental Figure S5C). Due to the low numbers of MM (*n* = 2) and VV (*n* = 5) patients, we restricted our longitudinal statistical modeling to the 17 MV cases. The mixed linear model showed that relative levels of plasma NT1-tau increased with disease progression (Figure 4C; MRC Scale predictor, β = −0.03, *p* = 0.008).

Collectively, our cross-sectional and longitudinal results indicate that plasma NT1-tau increases with functional decline and the rate of clinical progression in CJD, and the effect on rate of clinical progression is modified by the PRNP codon 129.

## 4. Discussion

In CSF from HC, AD, and CJD subjects, fragments of tau were found to be much more abundant that FL-tau. Although all forms of tau were increased in AD and CJD, levels of FL-tau accounted for less than 4% of MR-detected tau. The elevation of all forms of tau in AD CSF, in the absence of increased NfL, is consistent with our earlier finding that certain forms of tau are released from neurons independent of cell death. Moreover, it is notable that even in CJD, where there is severe and ongoing neurodegeneration, FL-tau was only 2.7 times higher than controls, whereas NT1-tau levels in CJD were more than 26-fold higher. Indeed, while the measurement of NT1 and MR-tau allowed for a near perfect separation of CJD from AD and controls, equal or superior to NfL, FL-tau performed less well. The modest change in FL-tau in CSF from individuals suffering with severe and chronic neurodegeneration suggests that FL-tau is not well released when axons degenerate and neurons die.

In cell culture experiments, we and others found that MR-tau is released from neurons independent of neuronal compromise [21,22,27], but treatment with excitotoxic levels of glutamate causes a massive increase in extracellular MR-tau coincident with an increase in markers of cell compromise [22]. In contrast, cultures decimated by glutamate treatment showed only a marginal increase in FL-tau [22]. These prior in vitro experiments, the results from the current study of CJD CSF, and recent studies of CSF from patients with a variety of neurodegenerative diseases [20,21,24,25,26,43] indicate that FL-tau is not readily released from either healthy or compromised neurons. Collectively, these results indicate that measurement of certain tau fragments will be more diagnostically useful than assessment of FL-tau.

Blood-based markers are more easily accessible and would facilitate the repetitive assessments essential for a longitudinal monitoring of disease onset in patients at risk for CJD, and as an objective read-out for clinical trials [15]. Given the incompatibility of MR-tau assays with GuHCl, and the high sensitivity and specificity of NT1-tau measurements in CSF, we focused our efforts on applying the NT1 assay to the analysis of CJD plasma. In initial experiments with plasma, we also used the FL assay. Both assays measured higher levels of tau in CJD plasma compared to controls and AD, but, as expected from the CSF results, the NT1 assay allowed for a better separation between the diagnostic groups than did FL-tau.

In plasma from control and AD subjects, the values measured by the FL and NT1 assays were of a similar magnitude. These results are consistent with our prior study, which revealed that the level of FL-tau relative to other forms of tau was much higher in plasma than in CSF [20]. This finding implies either a different half-life of certain forms of tau in CSF versus blood, or, more likely, a peripheral source of tau, which allows for a greater release of FL-tau than in brain. Whatever the reason for the relatively high levels of FL-tau in plasma, the better relationship between NT1-tau in plasma and CSF further emphasizes the greater potential of NT1-tau as a plasma biomarker for both AD and CJD. Moreover, it is noteworthy that several prior studies that used the Quanterix tau assay found interesting trends between plasma tau and the presence and/or severity of CJD. As in the NT1-tau assay, the Quanterix assay also relies on antibodies directed to the N-terminal domain of tau. However, our NT1-tau assay is superior to the Quanterix assay at discriminating AD and AD-MCI from controls [20,28], and allows for a perfect segregation of CJD from AD and controls in CSF.

Using the MRC Scale as a measure of functional decline, we investigated the relationship between plasma NT1-tau levels and the rate and stage of clinical progression in 145 patients with CJD. Plasma NT1-tau levels were associated with disease severity (MRC scale at the time of testing) and a faster rate of functional decline (assessed by MRC slope). Strikingly, the effect on functional decline was moderated by the *PRNP* codon 129 genotype. Plasma NT1-tau correlated with a faster rate of clinical decline in MM carriers (but not in VV and MV individuals). The MM genotype has a profound influence on the rate of functional decline in CJD, where a 10% functional loss on the MRC Scale is observed in only 5 days versus 12 days in VV, and 28 days in MV carriers [29]. Higher plasma tau levels have been reported in MM compared to VV and MV carriers [11,44,45], and prior studies using the Quanterix tau assay observed an interaction between the *PRNP* genotype and plasma tau levels and survival time [45,46,47]. Our results are in agreement with these previous findings, but we also show that plasma NT1-tau concentration correlated with a faster rate of clinical decline in MM.

As in our cross-sectional study, plasma NT1-tau was found to increase with disease progression in most cases (67%) available for longitudinal analysis. Notably, both MM cases and four out of five VV subjects showed increased plasma NT1-tau with advancing disease.

### Limitations

Although very promising, our study is not without limitations. The custom-made NT1-tau assay will need to be further developed from a research-grade to a clinical-grade assay. Future work will include a definition of reference materials and optimization of cut-offs before moving this biomarker to clinical routine or use in treatment trials. A further limitation is the predominance of patients with an MV genotype in our longitudinal study. New studies should include more MM and VV cases, and the overall number of patients and longitudinal timepoints should be increased. Finally, longitudinal studies of plasma NT1-tau (and additional forms of extracellular tau) in other tauopathies and across different disease stages are warranted to further elucidate the diagnostic and prognostic power of tau biomarkers across neurodegenerative diseases.

## 5. Conclusions

We provide evidence that plasma NT1-tau concentrations show promise as a minimally invasive biomarker for diagnosis and monitoring disease progression in CJD. Our results are particularly encouraging because, unlike CSF collection, which requires a lumbar puncture, blood samples can be easily and repeatedly obtained. Our findings warrant further investigation of NT1-tau as a predictor of disease course, its usefulness in clinical trial stratification, and its use as an outcome parameter in CJD trials.

## Data Availability

The data presented in this study are available from the corresponding author upon reasonable request. Data are not publicly available due to privacy issues as our research included individuals from rare neurodegenerative conditions.

## Supplementary Materials

The following are available online at https://www.mdpi.com/article/10.3390/cells10123514/s1, Supplemental Table S1: Participant Characteristics, Supplemental Figure S1: The in-house Simoa mid-region assay measures highly similar amounts of tau in CSF as the clinical-grade Innotest tau assay, Supplemental Figure S2: CSF NfL, NT1-tau, and mid-region tau allow for excellent discrimination of CJD from AD and controls, Supplemental Figure S3: Forms of tau in CSF measured with the mid-region tau assay are highly correlated with tau detected with the NT1-tau assay, but not the full-length tau assay, Supplemental Figure S4: Plasma NfL and NT1-tau, but not FL-tau, are elevated in AD subjects, Supplemental Figure S5: Plasma NT1-tau levels increase with disease progression of CJD.
