# Supplementary material for: NT1-Tau Is Increased in CSF and Plasma of CJD Patients, and Correlates with Disease Progression
Source: Cells. 2021 Dec 13;10(12):3514. doi: 10.3390/cells10123514 (PMC8700417; doi:10.3390/cells10123514)
Supplement: Supplementary file 1 [file cells-10-03514-s001.zip › cells-1480726-supplementary.pdf]

Table S1. Participant Characteristics

		CSF study cohort			Plasma study cohort 1*			Plasma study cohort 2	
		**Study 1**			**Study 2**			**Study 3**	**Study 4**
		CJD	AD	NC	CJD	AD	NC	CJD	
Total, No.		15	18	21	6	15	15	117	28
N with repeated measure		-	-	-	-	-	-	-	28
Mean number of repeated measures		-	-	-	-	-	-	-	2.7
Diagnosis	Definite CJD	2			2			93	
	Probable CJD	13			4			52	
CSF Aβ 1-42	Mean (SD)		491.8 (87.6)	985.8 (190.2)		469.9 (94.4)	1055.3 (194.3)		
CSF T-tau	Mean (SD)		923.2 (229.0)	264.5 (82.5)		692.5 (222.5)	295.1 (118.6)		
CSF T-tau/ Aβ 1-42 ratio	Mean (SD)		2.0 (0.8)	0.3 (0.1)		1.5 (0.4)	0.3 (0.1)		
PRNP codon 129	MM	9			3			44	
	MV	6			3			58	
	VV	0			0			43	
Age#	Mean (SD)	58.3 (10.6)	76.8 (6.6)	66.7 (11.4)	55.7 (14.5)	70.1 (7.7)	70.5 (5.3)	65.3 (9.0)	
	Range	42-76	65-86	51-80	42-76	53-80	65-81	25-83	
Gender	Female, n (%)	8 (53)	10 (56)	11 (52)	1 (17)	10(75)	10 (75)	75 (52)	
MRC scale#	Median (IQR)	14 (8-17)			16 (10-18)			8 (4-12)	
Onset to sample collection (days)	Median (IQR)	284 (160-337)			312 (140-340)			185 (108-334)	
Time until death (days)	Median (IQR)	126 (45-338)			139 (51-202)			42 (17-138)	

* For CJD patients, available plasma was taken from patients included in the CSF study cohort (*Study 1*), whereas plasma from AD and controls were from a separate study cohort (see "Material and Methods" for details); # at first sample collection

Mengel *et al*. Supplemental Figure S1

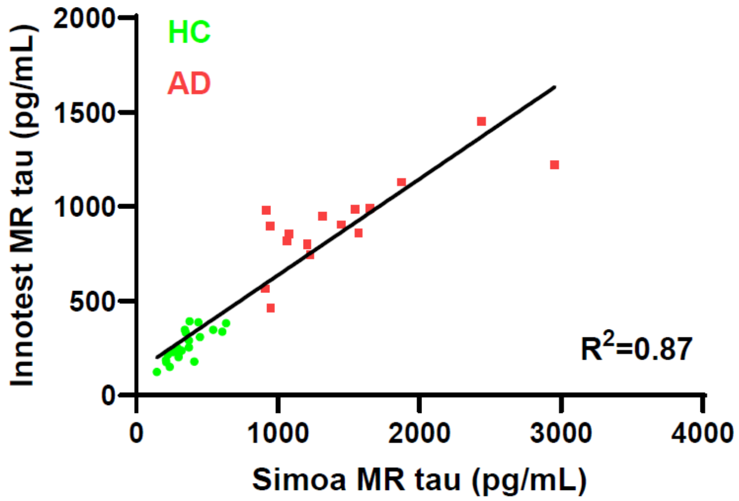

Figure S1. The in-house Simoa mid-region assay measures highly similar amounts of tau in CSF as the clinical-grade Innotest tau assay. CSF specimens from 21 AD (red squares) and 18 HC (green circles) (same subjects as in Figure 2) were analyzed using the Innotest tau assay (no GuHCl) and our in-house (in the presence of 0.063 M GuHCl) mid-region assay. The amounts of tau detected by both these mid-region assays were highly correlated (R^2=0.87, p<0.0001).

Mengel *et al*. Supplemental Figure S2

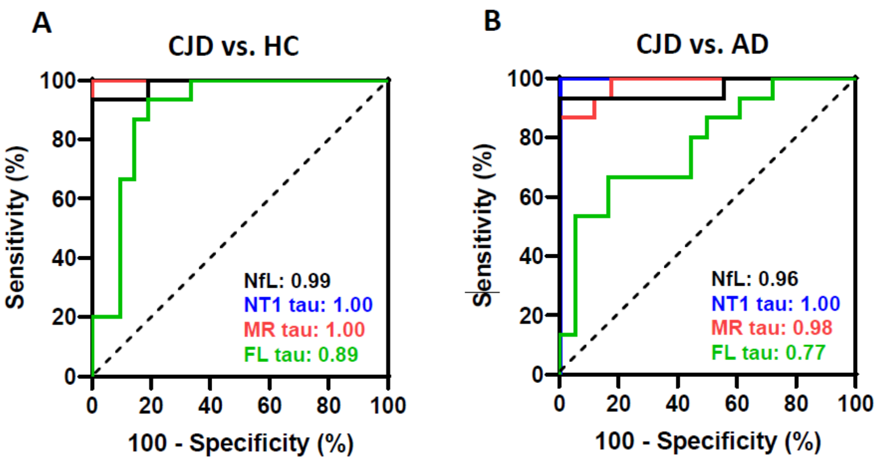

Figure S2. CSF NfL, NT1-tau, and mid-region tau allow excellent discrimination of CJD from AD and controls. As in Figure 2, *Study 1* CSF samples comprised 21 HC, 18 AD specimens collected in Gothenburg, and 15 CJD samples collected at UCL and were analyzed by the Simoa-based assays for NfL (black), NT1-tau (Tau12-BT2, blue), MR-tau (BT2-ADx202, red), and FL-tau (TauAB-Tau12, green). ROC curves of CSF NfL, NT1-tau, and MR-tau distinguish **(A)** CJD from HC, and **(B)** CJD from AD with excellent diagnostic certainty (AUCs ≥ 0.96). In comparison, there was reduced, but still good separation of **(A)** CJD and HC, and **(B)** CJD and AD with the FL-tau assay (AUCs = 0.89 and 0.77, respectively). Area under the curves (AUC) for ROC analyses were calculated using a non-parametric approach.

Mengel *et al*. Supplemental Figure S3

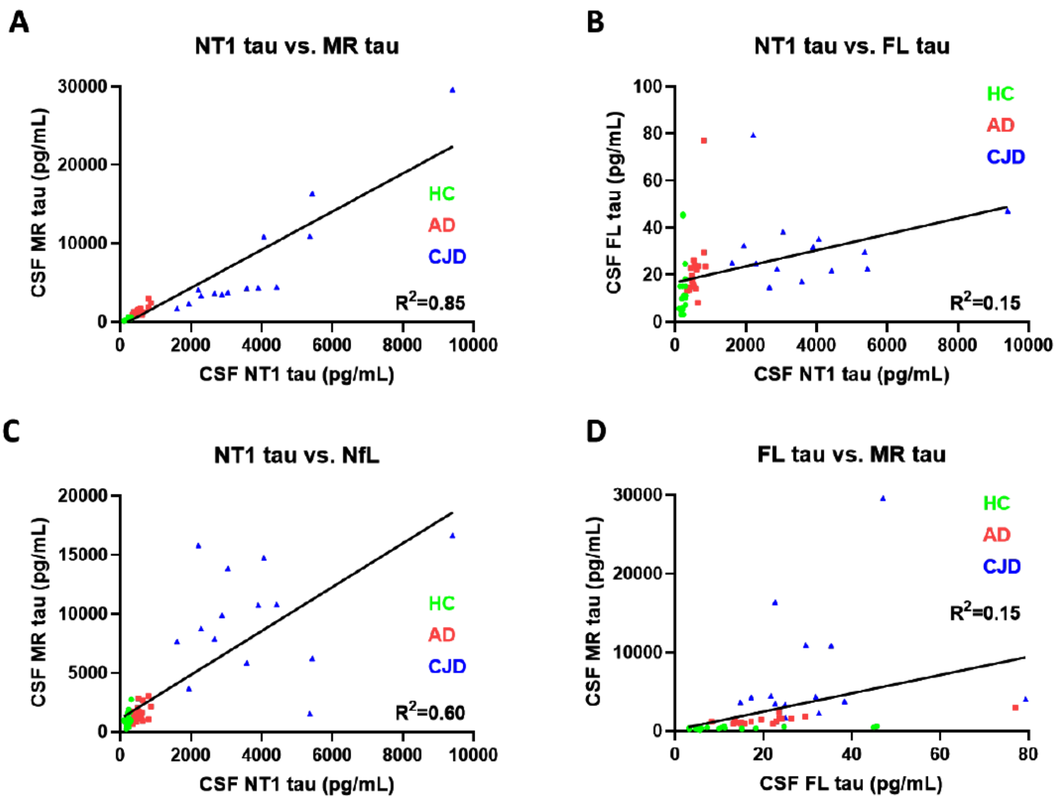

Figure S3. Forms of tau in CSF measured with the mid-region tau assay, are highly correlated with tau detected with the NT1-tau assay, but not the full-length tau assay. Linear regression analysis of results from *Study 1* CSF samples (21 HC - green circles), 18 AD - red squares, and 15 CJD - blue triangles) of **(A)** NT1-tau versus MR-tau, **(B)** NT1-tau versus FL, **(C)** NT1-tau versus NfL, and **(D)** FL-tau versus MR-tau. Significant associations were detected between **(A)** NT1 and MR-tau, and **(C)** NT1-tau and NfL, but not for NT1 and FL-tau, or FL and MR-tau.

Mengel *et al*. Supplemental Figure S4

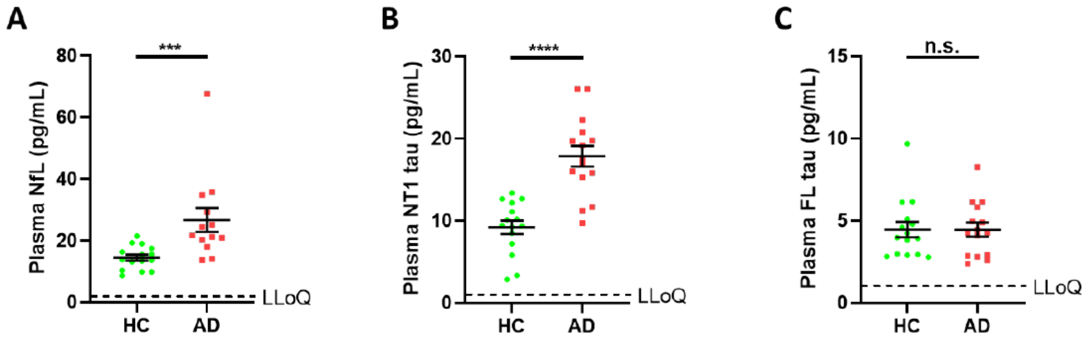

Figure S4. Plasma NfL and NT1, but not FL-tau are elevated in AD subjects. *Study 2* plasma specimens from HC (n= 15) and AD (n = 15) subjects were analyzed without addition of GuHCl using Simoa-based assays for **(A)** NfL, **(B)** NT1-tau, and **(C)** FL-tau. Each point represents a single individual and means ± SEM are indicated. Differences between groups were assessed with Kruskal-Wallis H test followed by Dunn's post-hoc test. Plasma **(A)** NfL, and **(B)** NT1-tau, but not **(C)** FL-tau were elevated in AD versus controls. n.s. non-significant; ***p<0.001; ****p<0.0001.

Mengel *et al*. Supplemental Figure S5

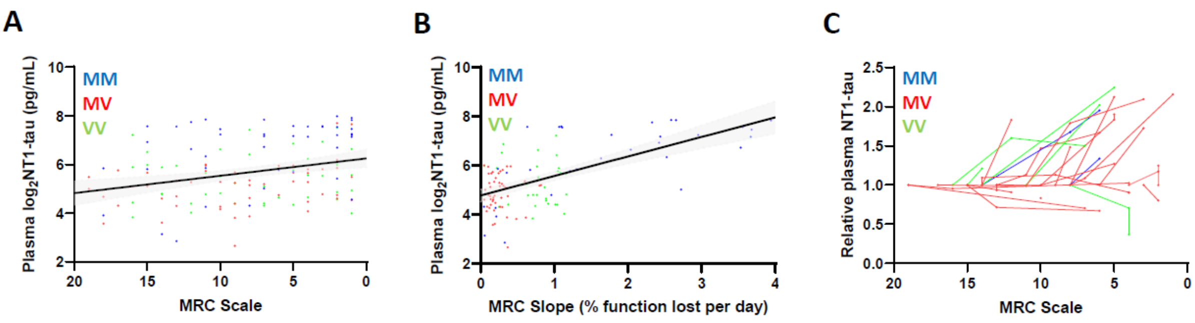

Figure S5. Plasma NT1-tau levels increase with disease progression of CJD. Correlation of plasma NT1-tau levels with **(A)** severity of functional impairment measured with the MRC Scale, and **(B)** the rate of clinical progression determined with the MRC Slope were calculated using linear regression in CJD patients from *Study 3* and baseline samples from *Study 4* (n=145). **(A)** Severity of functional impairment (lower MRC Scale) and **(B)** the rate of clinical progression correlated with higher plasma log_2NT1-tau levels. Symbols denote single measurements from MM (blue), MV (red) and VV (green) subjects. **(C)** Repeated plasma samples were collected from 2 MM, 17 MV, and 5 VV cases, and analyzed with the NT1-tau assay (*Study 4*, total number of specimens n= 63). Spaghetti plots show that repeated measures change with disease progression measured with the MRC scale in MM (blue), MV (red), and VV cases (green).
